# Human rights mechanisms for anti-corruption, transparency and accountability: enabling the right to health

**DOI:** 10.1080/16549716.2019.1699343

**Published:** 2020-03-20

**Authors:** Sharifah Sekalala, Haleema Masud, Rebekah Thomas Bosco

**Affiliations:** a University of Warwick, Coventry, UK; b Warwick Medical School, Coventry, UK; c WHO, Geneva, Switzerland

**Keywords:** Corruption, human rights, human rights mechanisms, health sector, accountability

## Abstract

**Background**: The presence of corruption in State institutions and broader society presents a significant obstacle to the right to the enjoyment of the highest attainable standard of health. The Universal Periodic Review, a Member State-led peer review system administered by the Human Rights Council, is a core tool of human rights, including the right to health accountability. This paper builds on existing research to examine processes that support State engagement on the issue of corruption. We identify opportunities for States to use the Universal Periodic Review to support anti-corruption, transparency and accountability to control corruption in the health-care sector.

**Objectives**: This paper focuses on health sector how human rights mechanisms, and particularly the Universal Periodic Review, can be a tool for greater accountability for the right to health for corruption in the health sector.

**Methods**: The research team applied qualitative content analysis methods to analyze all 135 Universal Periodic Review documents produced during 2018 in order to analyze how human rights mechanisms address the impact of corruption on the realization of the right to health.

**Results**: Although health rights violations are often addressed within human rights mechanisms such as the UPR, corruption remains under-addressed, suggesting that there are gaps in understanding how corruption can seriously undermine the right to health.

**Conclusion**: Human rights mechanisms should drive greater attention to the importance of addressing corruption in health. In order to make the UPR more effective, this paper suggests that there is a need to generate more awareness of corruption-based violations of the right to health in order to promote greater health accountabilityPractical tools such as strategic litigation and social audits can also contribute to creating greater transparency and accountability in dealing with corruption.

## Background

The World Health Organization (WHO) Constitution defines the right to health as the right to the highest attainable standard of physical and mental well-being [1]. The right to health is a legally-binding obligation, enshrined in international treaties, including the International Covenant on Economic, Social and Cultural Rights (ICESCR), the International Convention on the Elimination of All Forms of Racial Discrimination (ICERD), the Convention on the Elimination of All Forms of Discrimination against Women (CEDAW), the Convention on the Rights of the Child (CRC), the Convention on the Rights of Persons with Disabilities (CRPD), and the International Convention on the Protection of the Rights of All Migrant Workers and Members of Their Families. These treaties require States to respect, protect and fulfil the right to health, complementing and enhancing regional human rights treaties and domestic obligations.

Corruption is generally defined as the ‘misuse of public power for private gain’ [2]. Corruption can devastate good governance, the rule of law, development, and the equitable enjoyment of all human rights; notably, the right to health. In health, corruption can take many forms, as described by Vian and Crable in the International Encyclopedia of Public Health (Table 1) [3]. Corruption is often divided into ‘grand’ and ‘petty’ corruption, with the former referring to acts at a high level of government, distorting centralized policy, and the latter referring to smaller-scale corruption involving low and mid-level public officials [2]. Other distinctions are made between political and institutional corruption: political corruption refers to an act (such as the manipulation of policies and procedures) carried out by political decision-makers, whereas institutional corruption refers to the exploitation of institutional positions to influence processes and actions within institutions, leading to behavior which becomes normalized within the institution itself [2]. Corruption in health can be widespread (Table 1). Though research on the impact of corruption on specific health outcomes is limited, the UN Special Rapporteur on the right to health suggests that States with higher levels of corruption have a higher prevalence of poor health, including higher levels of infant and child mortality [4,5]. Furthermore, where vulnerabilities intersect – for instance, women in rural areas – the effect of corruption upon the provision of health may intensify [6]. While the focus of this research is corruption within the public sector, private sector ramifications are acknowledged, particularly in light of many models of healthcare service provision.

**Table 1.** Types of corruption in the health sector [3].

Area of process	Types of corruption and problems	Results
Construction and rehabilitation of health facilities	Bribes, kickbacks and political considerations influencing the contracting process Contractors fail to perform and are not held accountable	High-cost, low-quality facilities and construction work. Construction investments influences by bribes may also lead to further waste if recurrent costs to operate facilities are inadequately financed Location of facilities that does not correspond to need, resulting in inequities in access Biased distribution of infrastructure favoring urban- and elite-focused services, high technology
Purchase of equipment and supplies including drugs	Bribes, kickbacks and political considerations influence specifications and winners of bids Collusion or bid rigging during procurement Lack of incentives to choose low-cost and high-quality suppliers Unethical drug promotion Suppliers fail to deliver and are not held accountable	High-cost, inappropriate, or duplicative drugs and equipment Irrational prescribing Substandard equipment and drugs Inequities due to inadequate funds left to provide for all needs
Distribution and use of drugs and supplies in service delivery	Theft (for personal use) or diversion (for private sector resale) of drugs and supplies at storage and distribution points Sale of drugs or supplies that were supposed to be free	Lower utilization Patients do not get proper treatment Patients must make informal payments to obtain drugs Interruption of treatment or incomplete treatment, leading to the development of antimicrobial resistance
Regulation of quality in products, services, facilities and professionals	Bribes to speed process or gain approval for drug registration, drug quality inspection, or certification of good manufacturing practices Bribes or political considerations influence results of inspections or suppress findings Biased application of sanitary regulations for restaurants, food production and cosmetics Biased application of accreditation, certification, or licensing procedures and standards	Subtherapeutic or fake drugs allowed on the market Marginal suppliers are allowed to continue participating in bids, getting government work Increased incidence of food poisoning Spread of infectious and communicable diseases Poor-quality facilities continue to function Incompetent or fake professionals continue to practice
Human resources management	Bribes to gain place in medical school or other training Bribed to obtain passing grades Political influence, nepotism in selection of candidates for training opportunities or positions Bribes or regular payoffs to obtain/maintain position in government health services or medical facilities	Incompetent professionals practicing medicine or working in health professions Loss of faith and freedom due to unfair system Poor resource allocation decisions due to inaccurate health expenditure data (doesn’t reflect payoffs to superiors, effectively a tax on salaries) Increased informal payments as health workers seek to finance required pay-offs to keep their job Violation of individual rights Patients who receive unnecessary or harmful treatment
Medical research	Pseudo trials funded by drug companies that are really designed for marketing purposes Misunderstanding of informed consent and other issues of adequate standards in developing countries	Violation of individual rights Biases and inequities in research Patients who receive unnecessary or harmful treatment
Financial management	Embezzlement of budget allocation Theft of user fee revenue False recording of revenue to inflate or obscure financial position from stockholder or analysts (affects private health firms) Billing or reimbursement fraud	Reduced availability of public health programs and government medical services Lower quality of care Bankruptcy and loss of entrusted resources Loss of state dollars to fraud
Service delivery	Doctors use public facilities and equipment to see private patients Diversion of patients to private practice or privately owned ancillary services Utilization that is not medically indicated, in order to maximize income Withholding of care that is medically indicated (to solicit bribes) Absenteeism and shirking Informal payments required from patients for services that were supposed to be free of charge	Government loses value of investments without adequate compensation Employees are not available to serve patients, leading to a lower volume of services and unmet needs, and higher unit costs for health services actually delivered Reduced utilization of services by patients who cannot pay Impoverishment as citizens borrow or sell assets to pay for health care Loss of citizen faith in government

From *Fighting Corruption in Developing Countries: Strategies and Analysis*, edited by Bertram I. Spector. Copyright © 2005 by Lynne Rienner Publishers, Inc. Used with permission of the publisher.

In order to achieve the meaningful realization of the right to health, trillions of dollars are spent annually, of which an estimated 10-25% is lost through corruption, preventing people from enjoying their right to health [7]. Anti-corruption, Transparency and Accountability (ACTA) measures in health systems are increasingly recognized as central components for health systems strengthening and upholding the right to health. Without realizing the ACTA measures, resources meant to deliver on health can go waste, thus compromising human lives [8]. States’ obligation to respect, protect and fulfil the right requires addressing corruption where it interferes with citizens’ enjoyment of health. States are legally bound to design and implement ACTA measures under the United Nations Convention Against Corruption (UNCAC). Whilst State parties bear the ultimate responsibility, they are not the only actors with responsibilities to realize the right to health – private actors must respect human rights, ensuring that they comply with national laws and regulations, including regarding corruption [9,10].

Many scholars have called for a human rights response to corruption [11–13] and have identified a causal relationship between corruption and violations of the right to health [4,14]. However, the link between corruption and human rights violations is bidirectional: human rights violations and deficits create opportunities for corruption, and corruption results in human rights violations. Lack of participation and transparency, and violations of the right to a fair trial, the right to an effective remedy, and the right to information, among others, create situations where corruption is enabled, and may become normative within society [11,12].

Corruption has a high impact on human development; its broad effects include inappropriate legislation, policy, clinical practices and priorities; denied or delayed access to healthcare; and the loss of trust in facilities, personnel and governance. The specific costs of corruption are difficult to quantify; however, the negative impact on the right to health generally, as well as on the UN Sustainable Development Goals (SDGs) are clear [15]. Notably, Goal 16 requires States to commit to ACTA measures through substantial reduction of corruption and bribery in all forms by 2030.

The UN started to formally recognize corruption as a global problem in 2000 by adopting resolution 55/61, and then repeated resolutions in 2001, 2002, which resulted in adoption of the first ever international treaty on corruption, the United Nations Convention Against Corruption (UNCAC) in 2003. In recent years, several United Nations human rights mechanisms have acknowledged the negative impacts of corruption on the enjoyment of human rights. In addition to the work of the UN Special Rapporteur on the right to health, the UN Committee on Economic, Social, and Cultural Rights made 75 explicit references to corruption in their recommendations to States as a barrier to the fulfilment of rights protected under the Covenant including the right to health [see, for example, 16–26]. The Committee on the Rights of the Child has made 102 explicit references to corruption (the majority very general) as an impediment to the realization of rights protected within the Convention on the Rights of the Child, with some explicit references to corruption in the health sector or as an impediment to health-related rights [27–30]. Similarly, the Committee on the Elimination of Discrimination Against Women and the Committee on Migrant Workers have raised the issue of corruption in relation to violence, a harmful and important determinant of health [31,32].

In 2003, the former Sub-Commission on the Promotion and Protection of Human Rights appointed a Special Rapporteur on corruption and its impact on the full enjoyment of human rights. In her reports, she highlighted the general effects of corruption on civil and political rights, as well as economic, social and cultural rights [33–36]. She made specific reference to broad, negative ramifications to health and its underlying determinants [34 (Paragraphs 6, 13, and 21(d)), 35 (Paragraphs 11, 12, 34, 35 and 40)]. The Advisory Committee on the negative impact of corruption on the enjoyment of human rights succeeded the Special Rapporteur in the Human Rights Council, and their final report in 2015 highlighted the relationship between corruption and violations of the right to health [36]. The committee established that linking corruption and human rights creates new opportunities for monitoring and litigation [36].

In 2017, the Human Rights Council adopted a resolution which outlined the frameworks and mechanisms available to address corruption’s negative impact on human rights [37]. The resolution reminded States of their obligations and commitments, underlined stakeholder cooperation and coordination at national, regional and international levels, and invited the Office of the High Commissioner for Human Rights (OHCHR) and UNODC to coordinate their work to deepen understanding of the nexus between corruption and human rights, encouraging the Human Rights Council mechanisms to consider the issue of corruption within the respective mandates. There is a growing literature on the role and effectiveness of human rights mechanisms in promoting human rights and protecting the rights of citizens, specifically the right to health [38–40]. For instance, Gensen reviewed 169 research publications which illustrated the impact of National Human Rights Institutions [41]. However, these studies did not focus on how human rights mechanisms can be used to deal with corruption in the health sector.

## Objectives

Building on the relationship between corruption in the health sector and the right to health, this article focuses on how human rights mechanisms, particularly the Universal Periodic Review (UPR), can be used as tools for holding State and non-State actors accountable for the impact of corruption on the right to health.

## Method

The UPR was established by General Assembly Resolution 60/251 to review the human rights records of all UN Member States. The UPR is a State-driven process, led by the Human Rights Council (HRC). It provides the opportunity for each State to declare what actions they have taken to fulfil human rights obligations in their respective states. Acting as the only universal mechanism of this type, the UPR reviews human rights records of each state every four and a half years. Review takes place in three sessions every year, each session taking 2 weeks and reviewing up to 16 States, thus completing 192 states in one cycle. To date, two cycles of the UPR (2008–2011, sessions 1–12, and 2012–2016, sessions 13 to 26) have been completed and the third one is underway (2017–2021, sessions 14–40). The goal is to ensure that States are accountable for human rights violations. We were interested in analyzing whether there was any evidence of States curbing corruption in the health sector and recognizing it as a human rights violation.

The UPR process has four phases: (i) gathering and collating information; (ii) ‘interactive dialogue’ in the UPR Working Group, leading to an Outcome Report; (iii) final adoption of the Outcome Report, including recommendations to the reviewed state, which may be accepted, partially accepted, noted, or taken under further consideration; and (iv) follow up to the review process. The information gathered during phase I consists of a report submitted by the State under review (the ‘national report’); data gleaned from the reports of independent human rights experts and groups, such as the Special Procedures, human rights treaty bodies, and other UN entities; and information from other stakeholders, including national human rights institutions and non-governmental organizations. These data sources are then reviewed during phase II, through an interactive discussion between the State under review and other UN Member States. During this discussion, any UN Member State can pose questions, comments and/or make recommendations to the States under review. Following the review, a report is prepared (outcome report) that provides a summary of the actual discussion. In phase III the state under review can adopt the Outcome Report, which includes accepting, partially accepting, noting, or taking under further consideration the recommendations; and the fourth phase is concerned with the follow-up to the review during next session, where the state is required to submit a report about the actions that they have taken as a result of the recommendations.

In January 2018, the 29th session of the UPR was completed with 14 states (Bahamas, Barbados, Botswana, Burundi, France, Israel, Lichtenstein, Luxembourg, Mali, Montenegro, Romania, Serbia, Tonga and UAE). The dataset for this analysis consisted of the 142 documents (1196 pages) covering these 14 states. We chose this session because it was the most recent data available at the time of analysis. Although a non-random sample of countries, this dataset can give insight into the most-recent types of issues and concerns raised.

The documents were downloaded from the respective states’ UPR websites, and included the National Report; the Compilation of UN Information (based on information contained in the reports of the Special Procedures, human rights treaty bodies like the Committee on Economic, Social and Cultural Rights [CESCR], and other UN entities); the Annex to the Compilation; the Summary of Stakeholders’ Information; Questions submitted in advance and Addenda; and the Outcome of the Review, as well as any Addenda [42]. We searched all 142 documents for any text relating to corruption, using the search term ‘corruption’ and found a total of 206 occurrences. We read the paragraph containing each of these occurrences and looked to see whether each of these references to health corruption was translated into a formal recommendation in the Outcome Report. If it was, we extracted and summarized this recommendation.

We further analyzed all 2632 UPR recommendations made for all 14 countries in order to identify which health-related issues were mentioned and the type of actions demanded from the states under review. We classified each recommendation using content analysis by deductive coding, using the 21 codes given in the report on advancing the right to health through the universal periodic review prepared by the WHO and Human Rights Centre Clinic, University of Essex [43]. We used these 21 pre-existing codes to classify recommendations based on health issues highlighted [43], and also categorized the recommendations into six different types (see Table 2).

**Table 2.** 

No.	Type of recommendations	Explanation
1	International human rights mechanisms	Covers recommendations that encourage states to ratify international human rights treaties; invite UN Special Rapporteurs; implement recommendations from treaty bodies’ concluding observations; implement comments or other relevant documents.
2	Legislation	Covers recommendations that approve or call for changes in legislation; changes to the legal framework; the repeal of certain legal provisions.
3	National funding	Covers recommendations to allocate or increase funds to a certain issue that engages the right to health; the health sector or health services.
4	International cooperation (funding and technical assistance)	Covers recommendations that engage the international community, assistance, cooperation and funding, either by encouraging the State under review to seek assistance from other states, or by requesting the State under review to share its expertise in a particular region.
5	Policies and programmes	Covers recommendations concerned with the enforcement or implementation of human rights through policies, procedures, programmes, services or other facilities.
6	Other	Covers recommendations that refer to issues of health but do not fit any of the above categories.

From WHO and Human Rights Centre Clinic, University of Essex (permissions pending).

We then transferred all the recommendations to an excel spreadsheet. Each recommendation was considered as a unit of analysis for the purpose of assigning codes and to classify into different categories. It was possible for one recommendation to be assigned multiple codes, based on the issues highlighted in the contents of that recommendation. Once the codes were assigned to each recommendation, we counted the frequency of each code. We mainly chose this frequency count approach to facilitate comparison of the extent to which ‘corruption in health sector’ is discussed relative to the ‘other 21 categories of health-related topics’. However, to overcome the limitations of the frequency counts and deductive coding approach, we supplemented the analysis with a detailed account of corruption-related data in the UPR documents, and summarized where and in what context corruption was mentioned.

### Methodological limitations

Clearly, a simple search for the single word ‘corruption’ is a crude means of locating sections of the reports which address issues relating to corruption. Frequency counts may give some indication of the extent to which a report concerns itself with corruption, but this is only a very approximate measure. Frequency counts of health sector corruption relative to the other health-related issues raised during the UPR session may, however, give some idea of how extensively it is being addressed. Given the length of the combined set of reports, we did not have the capacity to read for understanding every report. However, we did read in detail each paragraph in which the word ‘corruption’ was found, in order to identify the context in which corruption was being discussed. We do of course note that there may have been other relevant paragraphs, not containing the word ‘corruption’, which might have contained information useful to our findings, and which we did not locate.

It is also important to stress that content analysis only examines the contents of the UPR documents, and does not illuminate how these might affect the actions of countries on corruption later. This would be an important area for future research, as would extending our study beyond the sample of 14 countries examined here. However, this study provides a cross-section of the latest data to give an insight into the way the UPR is addressing health sector corruption relative to other right-to-health related issues. This information could be useful when reviewing the role of the UPR process in dealing with corruption as a human rights issue for a larger sample of countries.

## Results

**Figure 1.**
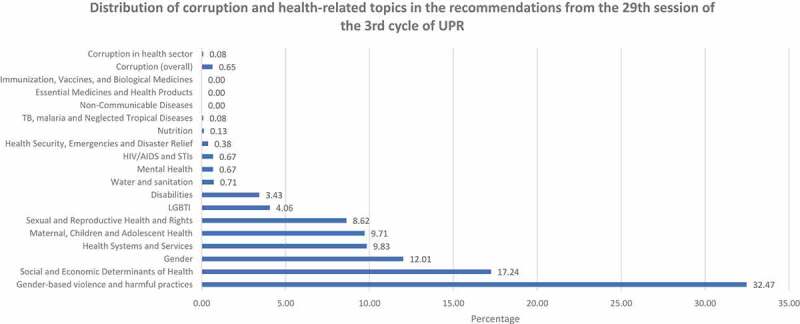
Distribution of corruption and health-related topics in the recommendations from the 29th session of the third cycle of UPR (January 2018).

**Figure 2.**
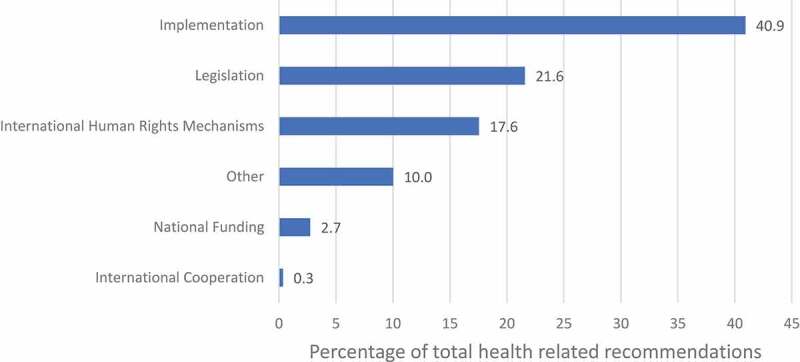
Types of health-related recommendations in the 29th session of the third cycle of the UPR (January 2018) [recommendations could be classified in more than one type].

Despite significant State engagement with the right to health, our analysis revealed a lack of focus on how corruption impacts the realization of the right. For example, of 2632 recommendations made during the 29th session, 1168 (44%) were health-related, but only 2 (0.08%) focused on corruption in health sector (see Figure 1). Seventeen recommendations (0.65%) were related to corruption in general (including the health-related corruption recommendations). France received the most recommendations, 297 (11.3%) in this session, followed by Burundi (237) and UAE (232). Barbados received the highest proportion of health-related recommendations, 76.6% (105 out of 137 recommendations made to the country) followed by Bahamas (69.5%) and Botswana (62.3%). States mostly made implementation-related recommendations (41%), followed by recommendations to ratify international human rights mechanisms (18%), and recommendations to enact relevant legislation (22%) (see Figure 2).

**Figure 3.**
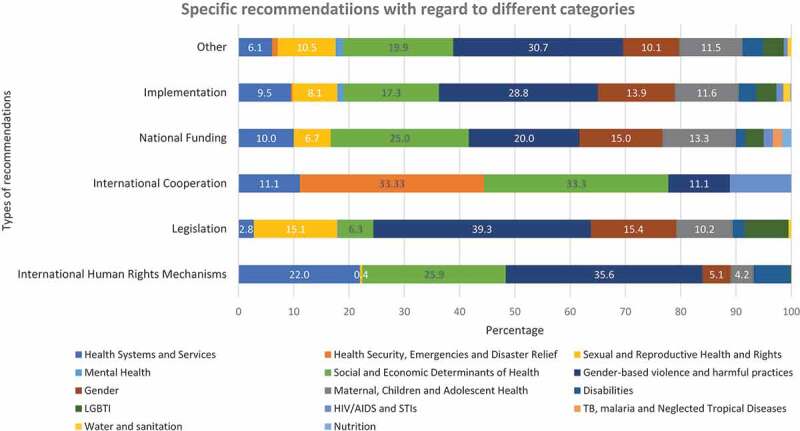
Distribution of specific health-related issues in the recommendations in the 29th session of the third cycle of the UPR. LGBTI: Health of Lesbian, Gay, Bisexual, Transgender or Intersex persons; STIs: Sexually Transmitted Infections; TB: Tuberculosis.

We further studied the distribution of specific health-related issues for all six categories of recommendations (Figure 3) and noted that for the most common category (implementation), the majority of recommendations were made for the issue of gender-based violence and harmful practices (28.8%). The corruption-related recommendations were mainly demanding implementation of policies and programs to combat corruption (12, 60%) from the States under review, followed by legislative changes and improvements (5, 25%) and others (3, 15%) demanding general non-specific anti-corruption actions or changes in training and curriculum of judiciary.

## Analysis of ‘corruption’ in the UPR

All the national reports mentioned corruption in reference to their efforts, progress or achievements to combat the issue, while the stakeholders’ reports mentioned it to highlight the concern for corruption and impunity for four states and to give recommendations mainly to increase transparency to combat corruption for two countries. The *Compilation of UN Information* reported corruption concerns for only two countries – Romania (high-level corruption impacting economic, social and cultural rights) and Montenegro (health sector corruption) – and this was in the reports of the CESCR, while UNHCR also commended the anti-corruption efforts of Liechtenstein. Questions submitted in advance were mainly for an update of the progress of the states under review. The interactive dialogue on the Outcome Report the focus narrowed to only seven countries. This dialogue consisted mainly on commending, acknowledging or noting the efforts of the state under review by other countries (three states), or the states’ responses about their progress (four states). For instance, for Montenegro, concern was shown about highly prevalent corrupt practices in health, education and employment sectors and the country was asked to take further measures.

A total of 17 corruption-related recommendations were made for six countries (Table 3). Montenegro and Romania both received six such recommendations while Serbia received two. Twelve countries made these recommendations (France made 3, Australia, Canada, and the USA each made 2, and single one by Germany, Norway, Korea, Estonia, Sweden, Azerbaijan, Ecuador, Portugal). Seven recommendations specifically demanded actions for corruption in judicial system (introducing and strengthening judicial reforms); eight recommendations were general about anti-corruption actions, i.e., asking states to implement laws and intensify efforts; one recommendation was about the need for legislation related to private sector corruption, two recommendations were focusing on combating corruption in health sector, while one recommendation demanded rejecting the legislation weakening anti-corruption efforts. Out of the 17 corruption-related recommendations made during the UPR session, 15 were supported by the states under review illustrating that States broadly accept recommendations on corruption when issued.

**Table 3.** Discussion of corruption in the UPR reports.

Country	National report	Compilation of UN information	Summary of stakeholders submissions	Questions submitted in advance	Outcome report		
					Summary of national report	Interactive dialogue	Recommendations
Bahamas	X	-	-	-	X	-	-
Barbados	X	-	-	-	X	-	X
Botswana	X	-	-	-	-	-	-
Burundi	X	-	X	-	-	X	X
France	-	-	-	-	-	-	-
Israel	-	-	-	-	-	-	-
Liechtenstein	X	X	X	-	-	-	X
Luxembourg	-	-	-	X	-	X	-
Mali	X	-	X	-	-	-	-
Montenegro	X	X	X	X	X	X	X
Romania	X	X	-	X	-	X	X
Serbia	X	-	X	-	X	X	X
Tonga	X	-	X	-	-	-	-
UAE	X	-	-	-	-	-	-
Total	11	3	6	3	4	5	6

X indicates a mention of corruption for one or more times in the relevant reports (sections).

## Corruption in the health sector

Corruption in the health sector was discussed in the UPR process for two States of the 14 reviewed (Romania and Montenegro) but was included in the recommendations of only one State, Romania in recommendations made by France and Korea. The CESCR raised concerns over corruption in the health sector, mainly in the form of informal user fees (Romania and Montenegro) and procurement (Montenegro). However, the resulting recommendation by Korea focused mainly on general health-sector corruption: ‘take measures to combat corruption in the health sector’. The CESCR highlighted that corruption adversely affects the full enjoyment of economic, social and cultural rights in Romania. The Committee noted low salaries for civil servants, health personnel and educators. They expressed concern regarding lenient penalties for corruption – highlighting noted risk factors for corruption in the sectors. The Special Rapporteur on extreme poverty called on Romania to fight corruption in the health sector so that non-official fees were not collected from patients. These concerns were highlighted in the recommendation made by France, ‘ … Continue to fight corruption in the heath sector, by taking both criminal action as well as measures to increase awareness of the negative effects of informal payments within the medical profession’.

Health sector corruption was also discussed in the case of Montenegro. The CESCR expressed concerns over informal payments provided by patients to health-care practitioners. The same committee also highlighted the issue of insufficient oversight of public procurement in the health-care sector. Australia also raised concerns about health-sector corruption in Montenegro, but it did not come up in the recommendations. The corruption-related recommendations for Montenegro were general ‘… Address corruption in the public sector and ensure the proper use of public authority in the managing and disposing of public property’.

Corruption, in general, was discussed in relation to six countries (Barbados, Burundi, Liechtenstein, Montenegro, Romania, and Serbia), resulting in a total of 15 recommendations (Table 4). In addition, human rights concerns in relation to acts or omissions of the private sector were discussed during the interactive dialogue for seven countries, resulting in a total of 16 recommendations. However, we contend that these low numbers, in comparison to the numbers of recommendations made regarding other categories of violations of the right to health, indicate a missed opportunity in addressing the issue of corruption in health, and its relationship to the right to health.

**Table 4.** Number of corruption and health-related topics in the recommendations of the 29th session of the third cycle of UPR.

	Recommendations				
	Country	Total	Health-related	Corruption in health sector	Corruption (General)
1	Bahamas	141	98	0	0
2	Barbados	137	105	0	1
3	Botswana	207	129	0	0
4	Burundi	237	99	0	1
5	France	297	112	0	0
6	Israel	240	51	0	0
7	Liechtenstein	126	40	0	1
8	Luxembourg	149	52	0	0
9	Mali	194	83	0	0
10	Montenegro	169	75	0	6
11	Romania	203	113	2	4
12	Serbia	190	67	0	2
13	Tonga	110	62	0	0
14	UAE	232	82	0	0
	Total	2632	1168	2	15

## Discussion

Our analysis suggests that the UPR mechanism is useful for identifying a variety of health-related human rights issues; however, it has been used only modestly to identify corruption in the health sector as a human rights concern. For example, of 2632 recommendations made during the 29th session, 1168 (44%) were health-related, but only 2 (0.08%) were focused on corruption in the health sector (see Figure 1). Furthermore, only 17 recommendations (0.65%) were related to corruption in general (including the health-related corruption recommendations). We found that even where recommendations were made, they were very general exhortations to ‘fight corruption.’ While this shows some consciousness of the problem, vague or general recommendations do not help target stakeholder action. It is important, therefore, to educate States and stakeholders about the ways in which the UPR review process can highlight corruption risks as a barrier to the right to health, and to identify more specific recommendations that might be helpful. For instance, the UPR process could be more specific and explicitly ask countries to make the procurement process open, transparent and free of corruption [44]. In the subsequent review, States would then submit specific measures they have taken in response. More state sensitization on this kind of specificity from the Special Rapporteur on the right to health would be welcome.

As we highlighted in the 'Background' section, the Special Rapporteur’s report is welcome in highlighting these linkages, and we recommend that the Special Rapporteur and other human rights bodies go further in raising the prominence of this issue by providing detailed guidance for Human rights bodies and States about this process. Additionally, specific guidance needs to be given to State parties and other stakeholders in order to highlight that corruption causes specific risks within the health sector and this undermines the realization of the right to health. Other human rights tools such as social audits, increased civil society litigation and strategic litigation can also strengthen accountability mechanisms within the UPR process as we discuss below.

### Social audits

Social audits are ways of measuring, understanding, reporting and ultimately improving an organization’s social and ethical performance. These audits create greater accountability in the health sector by enabling stakeholders, including intended beneficiaries, to participate in improving health planning and delivery [44]. Social audits can review State records, determining whether reported expenditures reflect reality, actualizing the Alma-Ata Declaration’s principle of participatory healthcare, and instituting grass roots accountability to counter the loss of faith in governance stemming from corruption. Initiatives that try to improve transparency, such as the WHO list of Medicine Price Information Sources and the Pharmaceutical System Transparency and Accountability Assessment Tool, can further strengthen mechanisms at the domestic level. This may be through providing information regarding pricing, and institutionalizing good governance through capacity building, transparency and accountability [45]. The UPR mechanism could integrate social audit information in determining whether states are complying with their health obligations in identifying instances of corruption.

### Strengthening civil society

Strong civil society and specialist rights-based organizations can enable the UPR and other human rights mechanisms to deal more robustly with corruption. An engaged civil society can participate in the accountability process, including the submission of amicus briefs, shadow reports or other research-based submissions, engaging in the UPR, monitoring and evidencing State activities, and making recommendations.

### Strategic litigation

Strategic litigation aims to achieve systemic legislative change and strengthen human rights through test cases. This has the advantage of raising public awareness and the profile of the issue in question. Strategic Litigation can draw on the UPR mechanism in order to strengthen arguments about a lack of state accountability for curbing corruption and the resulting right to health violations. For example, in the Ugandan Case of Centre for Health Human Rights and Development and Others V Attorney General (Constitutional Appeal No 1 of 2013), the Supreme Court held that the constitutional court wrongfully dismissed a 2012 petition that argued that the Ugandan government should be held liable for failure to provide adequate maternity services. The two cases involved women who had died in labor because of absenteeism by health workers who were engaged in private clinics, and, in one case, an inability to buy essential medical supplies required by health-care workers, in contravention with government guidance [Centre for Health Human Rights and Ors V AG Const Appeal No 1 of 2013]. A recent Transparency International report based on data for 64 countries highlighted that a prevalence of bribery is one of the main causes of maternal mortality, regardless of how wealthy a country is and how much it invests in health [46]. This case led to much tighter supervision of health workers at the community level, maternal death audits in order to follow up the cause of death when women died, and greater community awareness about governments’ obligations for maternal health [47].

## Conclusion

This paper has shown that human rights mechanisms should drive greater attention to the importance of addressing corruption in health. Mechanisms such as the UPR, that are increasingly being widely endorsed and supported by member States, have significant potential in addressing corruption in health within countries, but this remains largely underutilized. A major constraint facing these mechanisms is the diversity of issues that are examined, each with different root causes and contextual factors. Human rights mechanisms should incorporate specific guidance and measures of where corruption is likely to occur, and effective strategies for prevention. Given the negative impact of corruption, there is a need to engage with civil society organizations, so that they can push corruption in health onto the agenda of human rights mechanisms. Practical tools, such as strengthening civil society, strategic litigation and social audits, can also contribute to creating greater transparency and accountability in dealing with corruption.

## Paper context

Corruption in health – whether grand or petty – is associated with poor health, resulting in estimated losses of 10–25% of health budgets. This paper offers a unique insight, based on an analysis of the recommendations from the Universal Periodic Review, into how human rights instruments can help quantify, expose and target corruption in health. The paper underscores the role of, civil society engagement, and greater community participation as critical to greater transparency.
